# Chromatin-wide and transcriptome profiling integration uncovers p38α MAPK as a global regulator of skeletal muscle differentiation

**DOI:** 10.1186/s13395-016-0074-x

**Published:** 2016-03-15

**Authors:** Jessica Segalés, Abul B. M. M. K. Islam, Roshan Kumar, Qi-Cai Liu, Pedro Sousa-Victor, F. Jeffrey Dilworth, Esteban Ballestar, Eusebio Perdiguero, Pura Muñoz-Cánoves

**Affiliations:** Department of Experimental and Health Sciences, Pompeu Fabra University (UPF), CIBER on Neurodegenerative diseases (CIBERNED), Barcelona, Spain; Department of Genetic Engineering and Biotechnology, University of Dhaka, Dhaka, 1000 Bangladesh; Wyss Institute for Biologically Inspired Engineering, Harvard University, Boston, MA 02115 USA; Sprott Center for Stem Cell Research, Ottawa Hospital Research Institute, Ottawa, ON K1H 8L6 Canada; Chromatin and Disease Group, Cancer Epigenetics and Biology Programme (PEBC), Bellvitge Biomedical Research Institute (IDIBELL), Barcelona, Spain; Institució Catalana de Recerca i Estudis Avançats (ICREA), Barcelona, Spain; Present address: Buck Institute for Research on Aging, Novato, CA USA

## Abstract

**Background:**

Extracellular stimuli induce gene expression responses through intracellular signaling mediators. The p38 signaling pathway is a paradigm of the mitogen-activated protein kinase (MAPK) family that, although originally identified as stress-response mediator, contributes to establishing stem cell differentiation fates. p38α is central for induction of the differentiation fate of the skeletal muscle stem cells (satellite cells) through not fully characterized mechanisms.

**Methods:**

To investigate the global gene transcription program regulated by p38α during satellite cell differentiation (myogenesis), and to specifically address whether this regulation occurs through direct action of p38α on gene promoters, we performed a combination of microarray gene expression and genome-wide binding analyses. For experimental robustness, two myogenic cellular systems with genetic and chemical loss of p38α function were used: (1) satellite cells derived from mice with muscle-specific deletion of p38α, and (2) the C2C12 murine myoblast cell line cultured in the absence or presence of the p38α/β inhibitor SB203580. Analyses were performed at cell proliferation and early differentiation stages.

**Results:**

We show that p38α binds to a large set of active promoters during the transition of myoblasts from proliferation to differentiation stages. p38α-bound promoters are enriched with binding motifs for several transcription factors, with Sp1, Tcf3/E47, Lef1, FoxO4, MyoD, and NFATc standing out in all experimental conditions. p38α association with chromatin correlates very well with high levels of transcription, in agreement with its classical function as an activator of myogenic differentiation. Interestingly, p38α also associates with genes repressed at the onset of differentiation, thus highlighting the relevance of p38-dependent chromatin regulation for transcriptional activation and repression during myogenesis.

**Conclusions:**

These results uncover p38α association and function on chromatin at novel classes of target genes during skeletal muscle cell differentiation. This is consistent with this MAPK isoform being a transcriptional regulator.

**Electronic supplementary material:**

The online version of this article (doi:10.1186/s13395-016-0074-x) contains supplementary material, which is available to authorized users.

## Background

Cellular signaling is essential for the cells’ ability to respond to the environment by integrating external cues to intracellular mediators and effectors. Activation of mitogen-activated protein kinases (MAPKs) constitutes a paradigm of intracellular signaling. p38, a subgroup of the MAPKs, was initially identified as a transducer of the response to inflammatory and environmental stress conditions. There are four p38 MAPKs in mammals: MAPK14 (p38_α_), MAPK11 (p38_β_), MAPK12 (p38_γ_), and MAPK13 (p38_δ_) [1, 2]. Activation of these MAPKs has also been associated with the differentiation capacity of several stem cell types. In particular, p38 plays an integral role in the fate decision of stem cells of the skeletal muscle lineage [3, 4]. Muscle stem cells (also called satellite cells), established early during development, are marked by the expression of the paired-box transcription factor Pax7, and have as principal mission sustaining skeletal muscle regeneration [5, 6]. When stimulated by an injury or disease, these normally quiescent stem cells are activated, begin to proliferate as myoblasts and, subsequently, they either exit the cell cycle, differentiate and fuse to form new fibers (or repair damaged ones), or self-renew to replenish the satellite cell pool. In vitro studies using cellular models (satellite cell-derived primary myoblasts or myoblast cell lines) that recapitulate the myogenic stages of the in vivo regeneration process, in combination with the chemical inhibitor of p38α/p38β SB203580, have shown an active participation of the p38 MAPK pathway in each stage, with a principal function as a regulator of the myoblast proliferation-to-differentiation transition, by inducing cell cycle withdrawal and expression of muscle differentiation-specific genes [3, 4, 7–9].

Consistent with their kinase activity, several transcription factors can be directly phosphorylated by p38α/β MAPKs, including E47, the dimerization partner of the master myogenic regulatory factors (MRFs) of the MyoD family, and MEF2, a transcription factor cooperating with the MRFs in myogenic gene transcription; these phosphorylation events have a profound effect on gene expression as they modulate the activity of MyoD-E47 and MEF2 on muscle-specific promoters [3, 10–14]. Furthermore, by phosphorylating the chromatin-associated protein BAF60c, p38α/β kinases contribute to the assembly of the myogenic transcriptosome on the chromatin of muscle loci by promoting the recruitment of SWI/SNF chromatin remodeling complex [15–17] and ASH2L-containing mixed-lineage leukemia (MLL) methyltransferase complex [18, 19]. Through phosphorylation, p38α also recruits SNF2-related CBP activator protein (SRCAP) subunit p18^Hamlet^ to muscle loci, which is in turn required for H2A.Z accumulation and transcriptional activation [20]. p38α-mediated phosphorylation of Ezh2, the enzymatic subunit of polycomb repressor complex 2 (PRC2) also regulates the expression of Pax7, thus controlling the decision of satellite cells to proliferate or differentiate [21]. By contrast, p38γ represses MyoD transcriptional activity by direct phosphorylation, via association with the H3K9 methyltransferase KMT1A, thereby also influencing this myogenic decision [22]. Thus, the p38 MAPK pathway can either activate or repress gene expression in satellite cells, depending on the engagement of specific p38 isoforms. Notably, in vitro studies using satellite cells lacking individual p38 family members showed that the four p38 isoforms are not completely redundant during myogenesis [7, 23, 24], and uncovered a predominant role of the p38α isoform in myogenic differentiation [7, 23, 24], with p38γ signaling contributing to proliferation by preventing premature differentiation [22], whereas p38β and p38δ appeared rather dispensable for these processes [7, 23]. Because muscle regeneration in vivo can proceed quite effectively in the absence of the p38γ isoform [23], but is defective in the absence of p38α [25], p38α is indeed acknowledged as the master kinase for reprogramming gene expression during the proliferation-to-differentiation switch of satellite cells in vitro and in vivo.

Studies in yeast have shown that the p38 MAPK homolog, Hog1, activates transcription in response to osmotic stress by acting directly at chromatin [26, 27]. Likewise, mammalian p38 MAPK can bind to some stress-responsive loci upon exposure to several types of stresses, emphasizing that the association of the MAPK with target promoters can stimulate gene expression [28]. In skeletal myoblasts, p38α has also been found to bind certain muscle-specific genes such as myogenin, muscle creatine kinase, and myosin heavy chain during the process of myogenic differentiation [15, 21]. However, whether p38α can regulate gene transcription globally during myogenesis via direct action on gene promoters is unknown. Here, we assessed the genome-wide enrichment of the p38α MAPK isoform, coupled to the p38α-dependent global gene expression program, during the switch of myogenic cells from proliferation-to-differentiation stages. Our findings enlarge the picture of p38α MAPK as an important coordinator of myogenic gene expression through direct association to chromatin.

## Methods

### Cell culture

The C2C12 myoblast cell line was cultured at 37 °C in 5 % CO2 in Dulbecco’s Modified Eagle’s Medium (DMEM; Invitrogen) with 10 % fetal bovine serum (FBS) and supplemented with antibiotics (100 U/ml penicillin and 100 μg/ml streptomycin (growth medium (GM)).

Satellite cells were obtained from p38α-deficient and WT mouse muscles, as described in [7] and their myoblast progeny was maintained on collagen-coated dishes in Ham’s F10 medium supplemented with 20 % FBS, 100 U/ml penicillin, 100 μg/ml streptomycin, and 5 ng/ml bFGF (GM). These cells are referred to as satellite cells throughout the study. Experiments were performed by plating cells on Matrigel™ (BD Biosciences) Basement Membrane Matrix-coated dishes. To induce differentiation in both primary and C2C12 myoblasts, GM was replaced by differentiation medium (DM) (DMEM supplemented with 2 % horse serum, 2 mM l-glutamine, 100 U/ml penicillin, and 100 μg/ml streptomycin) at myoblast subconfluence. For p38α/β MAPK inhibition studies, SB203580 was added directly to DM at a final concentration of 5 μM.

### Western blotting

Western blotting was performed as described previously [7]. Antibodies used were phospho-p38 (Thr180/Tyr182) (Cell Signaling 9211) and Tubulin (Sigma T-6199).

### Immunofluorescence in C2C12 myoblasts

C2C12 cells were fixed in PFA 4 % for 10 min at room temperature, washed with PBS, and permeabilized with 0.5 % Triton X-100 in PBS during 10 min. Cells were incubated with blocking solution (10 % goat serum, 10 % BSA in PBS) for 1 h at room temperature and then overnight at 4 °C with p38α antibody (Santa Cruz Biotechnology sc-535) diluted 1/50 in blocking solution. After three washes in PBS-Tween 0.025 %, cells were further incubated with goat anti-rabbit conjugated with Alexa-Fluor 488 (Invitrogen; 1/250) for 1 h. DAPI (1/1000) was used to label DNA, and the slides were mounted with Mowiol. Imaging was carried out with TCS-SPE confocal microscope (Leica) using LAS AF software.

### RNA isolation, reverse transcription (RT), and quantitative PCR (qPCR)

Total RNA from proliferating and differentiating myoblasts was isolated with TriPure Isolation Reagent and quantified with Nanodrop. M-MLV reverse transcriptase (Promega) was used to synthesize cDNAs from the total RNA (1 μg) following the manufacturer’s recommendations. RT-qPCR reactions were performed with SYBR Green in 384-well plates using the Roche LC-480 cycler (Roche Applied Science). The mRNA expression of target genes was normalized to L7 expression, and the data are represented as the mean ± SD of three independent experiments. Primer sequences are listed in Additional file 1: Table S2.

### Chromatin immunoprecipitation (ChIP) assay

ChIP assays were performed from approximately 5*10^6^ C2C12 cells per experiment. Briefly, cells were cross-linked with 1 % formaldehyde for 10 min at room temperature, and the crosslinking was then quenched with 0.125 M glycine for 5 min. Cross-linked cells were washed twice with cold PBS, resuspended in 600 μl of SDS lysis buffer (1 % SDS, 10 mM EDTA, 50 mM Tris-HCl, pH 8.1, plus protease inhibitors) and incubated on ice for 10 min. Cell lysate was sonicated to shear chromatin to an average length of 200–500 bp using a Diagenode Bioruptor. Sonicated samples were centrifuged at 13,000 rpm for 10 min and the chromatin concentration of the supernatant was quantified. Seventy microgram of chromatin were diluted 10-fold in ChIP dilution buffer (0.01 % SDS, 1.1 % Triton X-100, 1.2 mM EDTA, 167 mM NaCl, 16.7 mM Tris-HCl, pH 8.1, plus protease inhibitors) and immunoprecipitated overnight at 4 °C with 3 μg of p38α antibody (Cell Signaling 9218) and 1 μg of MyoD and Mef2 antibodies (Santa Cruz Biotechnology sc-304 and sc-13917, respectively) or control IgG antibody (Cell Signaling 2729). Twenty microliter of Magna ChIP Protein A + G Magnetic Beads (Millipore) were added and incubated for 1 h at 4 °C. Beads were successively washed with 1 ml of different buffers: low-salt immune complex wash buffer (0.1 % SDS, 1 % Triton X-100, 2 mM EDTA, 20 mM Tris-HCl, pH 8.1, 150 mM NaCl), high-salt immune complex wash buffer (0.1 % SDS, 1 % Triton X-100, 2 mM EDTA, 20 mM Tris-HCl, pH 8.1, 500 mM NaCl), LiCl immune complex wash buffer (0.25 M LiCl, 1 % NP40, 1 % deoxycholate, 1 mM EDTA, 10 mM Tris-HCl, pH 8.1), and TE (1 mM EDTA, 10 mM Tris-HCl, pH 8.0). To elute the immunocomplexes, beads were incubated twice in elution buffer (1 % SDS, 100 mM NaHCO3) for 15 min at room temperature. The crosslinking was reverted by further incubating the DNA at 65 °C overnight. DNA was extracted with phenol/chloroform, ethanol-precipitated and resuspended in 80 μl of H_2_O for further analysis. Bound fraction and input were analyzed by PCR and qPCR using specific primer sets for each of the analyzed promoters (see Additional file 1: Table S2). Relative recruitment is calculated as the amount of amplified DNA normalized to input and relative to values from control IgG immunoprecipitation, which were set as 1 in each calculation.

### Microarray analysis

RNA samples were collected from C2C12 cells (proliferating and differentiated for 24 h ± the p38α/β inhibitor SB203580) and primary myoblasts from WT and p38α-deficient mice in proliferation conditions and at 24 h of differentiation. Transcriptome analysis was performed using the 44K Whole-Mouse Genome (Agilent), and the RNA labeling and hybridization process was performed following the manufacturer’s instructions. Microarray analysis was performed with three samples per condition; for the primary myoblasts, each sample was a pool of three independent cultures. Moreover, and to avoid bias labeling artifacts, dye swap experiments were performed. Fluorescent images were obtained with an Agilent G2565BA scanner. Microarray images were quantified using GenePix software. Only spots with signal intensities twice above the local background, not saturated, and not flagged by GenePix were considered reliable and used for subsequent analysis. Extracted intensities were subtracted from the local background, and the log2 ratios were normalized in an intensity-dependent fashion by global LOWESS. For normalization and differential expression analysis, we used Bioconductor [29] (www.bioconductor.org) package LIMMA [27] for Agilent two-color microarray data. For differential expression analysis, we filtered for genes that show at least Log2FC 0.25 (≥+0.25 for upregulation and ≤−0.25 for downregulation).

### ChIP-on-chip analysis

Chromatin immunoprecipitation was performed in quadruplicate from independent C2C12 cultures of each condition. Each sample was cross-linked, and immunoprecipitation was performed as described above using p38α-specific antibodies (Cell Signaling). To avoid the high variability detected with p38α antibody, immunoprecipitated and input DNA were pooled, labeled by ligation-mediated PCR with Cy5 and Cy3 fluorescent dyes, respectively. Pools of labeled DNA were hybridized to a 244K Mouse Promoter Chip (Agilent) following the manufacturer’s instructions. The array was scanned and analyzed with GenePix software as explained above. Genome-wide location data were normalized, and the ratio of immunoprecipitated to input DNA was determined for each probe. For each array, a confidence value (*P* value) was calculated for each probe using an error model as explained before [27]. Briefly, probes that scored significantly above background were then combined into probe sets of three probes if they passed the following filter: the center probe in the probe set has a single point *P* value <0.001, one of the flanking probes has a single point *P* value <0.01, and the three probes combined have a *P* value <0.001. For binding peaks that typically span over transcribed regions and thus span over several probes, the following filter was used: the center probe in the probe set has a single point *P* value <0.005, the three probes combined have a *P* value <0.0001 and one of the flanking probes has a combined *P* value <0.001. Bound probe sets that overlapped were collapsed into bound regions. For each bound region, the closest ORF was then assigned and the maximum ChIP enrichment identified (see Additional file 2: Table S1).

### Bioinformatic and statistical analysis

Gene ontology analysis was performed using DAVID functional annotation clustering [30]. Gene set enrichment analysis (GSEA) was performed using the default parameters [31]. Venn diagrams were generated using The BEG Ugent tool (http://bioinformatics.psb.ugent.be/webtools/Venn/). Volcano plots of gene expression and statistical analysis data were performed using Graphpad Prism software. Quantitative data displayed as histograms are expressed as means ± standard error of the mean (represented as error bars). Results from each group were averaged and used to calculate descriptive statistics. Mann–Whitney *U* test (independent samples) was used for pairwise comparisons among groups at each time point. Statistical significance was set at a *P* value <0.05.

### Mice

Mice carrying the floxed p38α, allele were generated by Boehringer Ingelheim Pharmaceuticals Inc. (Ridgefield, USA). Floxed p38α mice were crossed to More-CRE knock-in mice to obtain p38α knockout mice. All animal experiments and isolation of satellite cells from wild type and knockout mice [7] were approved by the ethics committee of the Barcelona Biomedical Research Park (PRBB) and by the Catalan Government and used sex-, age- and weight matched littermate animals.

## Results

### Consequences of genetic and chemical p38α inhibition on the gene expression program of differentiating myoblasts

**Fig. 1:**
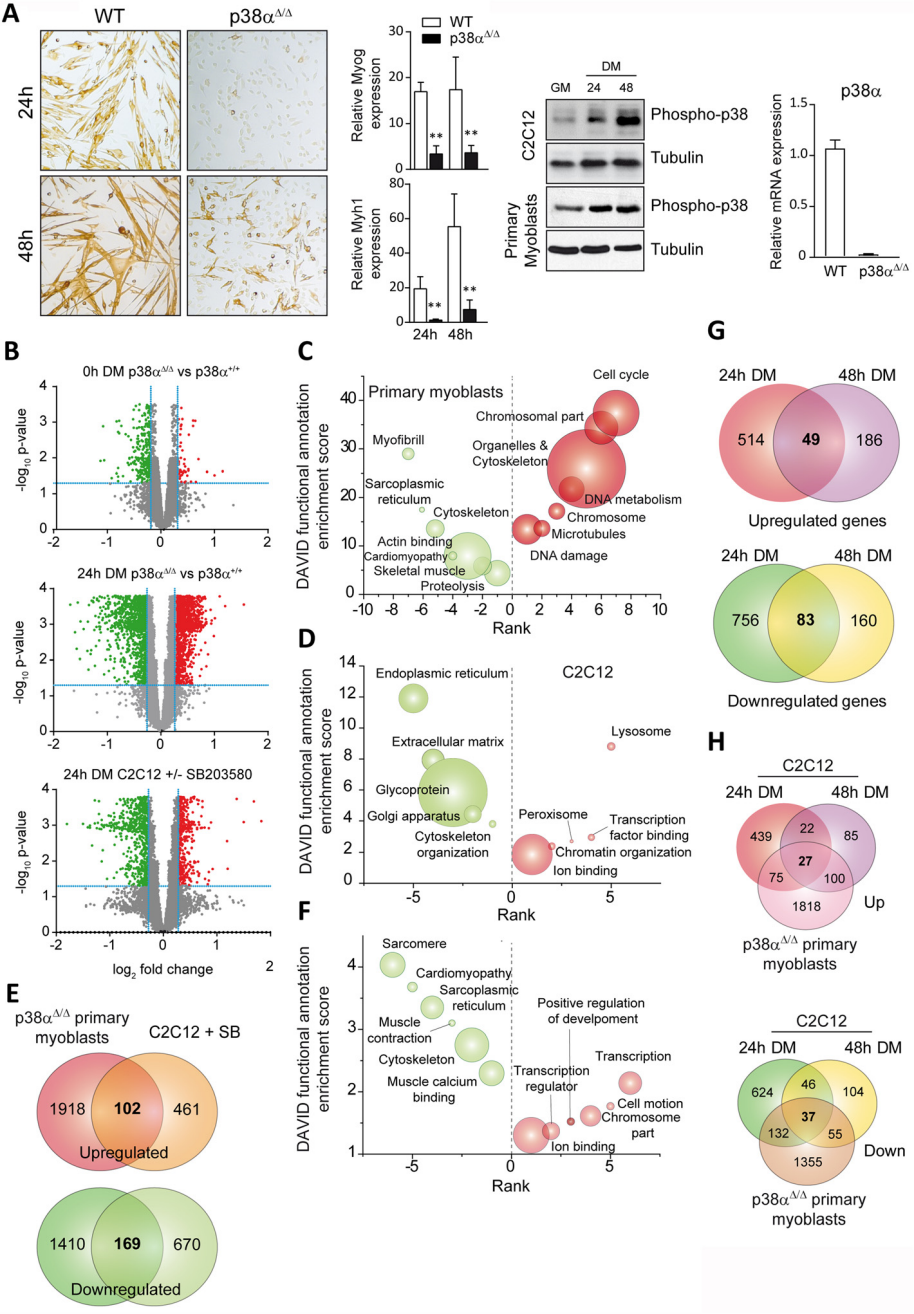
Gene expression analysis of p38α-deficient satellite cells and C2C12 cells treated with SB203580 (SB). **a** Satellite cells and C2C12 myoblasts were cultured in growth medium (GM) until subconfluent and then shifted to differentiation medium (DM) at the indicated time points. (*left*) Representative pictures of WT and p38α-deficient satellite cells 24 and 48 h in DM, and relative expression levels of differentiation-specific genes Myog and Myh1 analyzed by qPCR. (*right*) Activation of p38 was analyzed in both cells types by Western Blot using an anti-phospho-p38 antibody and expression of p38α in satellite cells derived from WT, and p38α-deficient mice was analyzed by qPCR (*right panel*). **b** Volcano plot showing differentially expressed genes in p38α-deficient versus WT satellite cells in GM, and DM for 24 h (*top and middle panel*, respectively) and in SB-treated C2C12 cells versus untreated (*bottom panel*). Downregulated and upregulated genes are marked in green and red, respectively. **c** Gene ontology (GO) analysis of differentially expressed genes in p38α-deficient satellite cells over WT at 24 h DM was performed using DAVID. Enriched GO annotations of downregulated and upregulated genes are shown in green and red, respectively. **d** As in (**c**) GO analysis of differentially expressed genes in C2C12 myoblasts (SB-treated versus untreated) at 24 h in DM. **e** Non-proportional 2-way Venn diagram comparing the effect in gene expression of p38α-deficient satellite cells at 24 h in DM (over WT) with the effect of chemical inhibition of p38α in C2C12 cells (SB treated over untreated). Only 102 and 169 genes are commonly upregulated and downregulated, respectively. **f** As in (**d**) GO analysis of genes that are commonly upregulated and downregulated in both C2C12 cells treated or not with SB and in satellite cells after 24 h in DM. **g** Comparison between expression arrays performed in C2C12 cells treated with SB203580 at 24 and 48 h in DM. The non-proportional 2-way Venn diagrams show the number of upregulated and downregulated genes in C2C12 cells treated with SB203580 over untreated cells. **h** Comparison between expression arrays performed in C2C12 cells treated with SB203580 (24 and 48 h DM) and p38α-deficient satellite cells

Because p38α controls the transition of myoblasts from proliferating to differentiating stages, we aimed to study specifically the changes in gene expression occurring during this transition (Fig. 1a). To increase the robustness of the experimental strategy, we used distinct myogenic cellular models and p38α-inhibiting alternatives: satellite cells obtained from muscle of wild type (WT) mice or mice with deletion in p38α [7], and the C2C12 myoblast cell line, cultured in the absence or presence of the p38α/β inhibitor SB203580 (SB) (Fig. 1a). Because the p38β isoform is not (or lowly) expressed in C2C12 myoblasts (Additional file 3: Figure S1), the inhibitory action of SB203580 on these cells will likely be attributable to p38α. Genome-wide microarrays of both types of cells (with/without genomic or chemical p38α-inhibitory conditions) in proliferation (growth medium (GM)) and early differentiation (24 h in differentiation medium (DM)) states were performed.

Through these analyses, we identified a large number of genes regulated by p38α (Fig. 1b; Additional file 2: Table S1), being this regulation particularly marked at the early 24 h myoblast differentiation stage. Gene ontology (GO) analysis using DAVID functional annotation clustering of the GO terms showed a downregulation in expression of muscle-differentiation-specific genes for satellite cells lacking p38α, whereas genes involved in proliferation and cell-cycle progression were upregulated (Fig. 1c). p38α signaling in satellite cells also appeared to regulate multiple genes and transcription factors that had not been previously related to myogenesis, for example, genes encoding signaling proteins and genes involved in proteolysis or DNA metabolism and damage. In contrast, SB treatment of C2C12 cells caused downregulation of genes related to secretory pathways and cytoskeleton organization, whereas the expression of genes related to transcription, chromatin organization, or ion binding was upregulated (Fig. 1d). Of note, the cell-cycle-related genes that were found upregulated in p38α-deficient satellite cells after 24 h in DM remained unchanged in SB-treated C2C12 cells at the same time point; however, SB-treated C2C12 cells showed upregulation of these proliferation-related genes at a later time point in DM (i.e., 48 h), according to the recently published data [8], suggesting a delay in the cell-cycle exit of C2C12 immortalized myoblasts, compared to primary satellite cells, in differentiation-promoting conditions. Of the p38α-regulated genes in both experimental conditions, only 102 and 169 genes were commonly up- and downregulated, respectively, in SB-treated C2C12 myoblasts and p38α-deficient satellite cells (Fig. 1e). Thus, the stringency imposed by the use of distinct muscle cell models and p38-inhibiting strategies allowed the identification of a robust list of bona fide genes dependent on p38α at the onset of myogenic differentiation (Additional file 2: Table S1). GO analysis of genes commonly regulated in SB-treated C2C12 cells, and p38α-deficient satellite cells at 24 h DM showed that the expression of muscle differentiation-specific genes is commonly downregulated in both types of cells in the absence of p38α signaling (Fig. 1f). In contrast, genes commonly upregulated in both conditions were related to the transcription and cell motion processes (Fig. 1f).

A further comparison of the expression arrays from C2C12 myoblasts in DM for 24 and 48 h (in the absence or presence of SB) revealed a dramatic difference in both time points: only 20 % of the genes upregulated by the 48 h DM + SB treatment were also upregulated at the earlier DM + SB 24 h time point (Fig. 1g); likewise, only 30 % of the downregulated genes at the 48 h DM + SB treatment were common with the 24 h DM + SB conditions. Thus, p38α regulates the muscle differentiation gene program in C2C12 myoblasts with distinct kinetics (i.e., p38α-dependent early and late myogenic genes) (Fig. 1g). Interestingly, comparison of the 24 and 48 h DM + SB C2C12 cells with the 24 h DM p38α-deficient satellite cells demonstrated that, in addition to cell-cycle exit, the kinetics of the p38α-dependent proliferation-to-differentiation gene expression transition program differ between both cell types; indeed, this transition was faster in satellite cells, as shown by the similar number of common p38α-regulated genes in the 24 h-DM satellite-cell time point and in C2C12 cells at both time points (Fig. 1h). It is probable that the faster differentiation kinetics might be related to the more rapid and pronounced activation of p38 MAPK signaling in satellite cells than C2C12 cells in the GM to DM transit conditions (Fig. 1a).

**Fig. 2:**
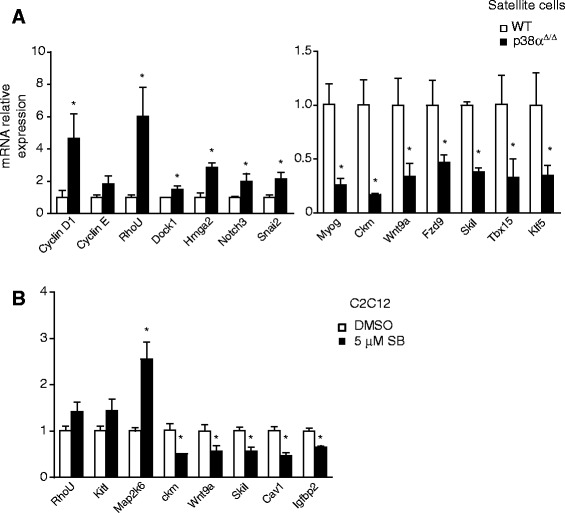
Validation of the p38α-dependent genes from expression arrays. **a** p38α-regulated genes were validated by qPCR in satellite cells derived from WT and p38α-deficient mouse muscles after 24 h in DM. **b** Genes regulated by chemical inhibition of p38α were validated by qPCR in differentiating C2C12 myoblasts (24 h DM) treated or not with SB. Different gene networks are regulated by p38α: muscle-specific and cell-cycle regulator genes, transcription factors, and genes encoding signaling proteins. Values are mean ± SEM of three independent experiments. Non-parametric Mann–Whitney *U* test was used for comparisons (*P* values <0.05)

We next performed qPCR analysis of p38α-deficient and WT satellite cells (Fig. 2a) and C2C12 myoblasts-treated or not with SB-treated (Fig. 2b) to validate the gene expression microarray results. We confirmed that transcripts belonging to different gene networks are regulated by p38α (in both genetic and chemical inhibitory settings), including muscle-specific and cell-cycle-regulatory genes, multiple transcription factors, and genes encoding signaling proteins.

**Fig. 3:**
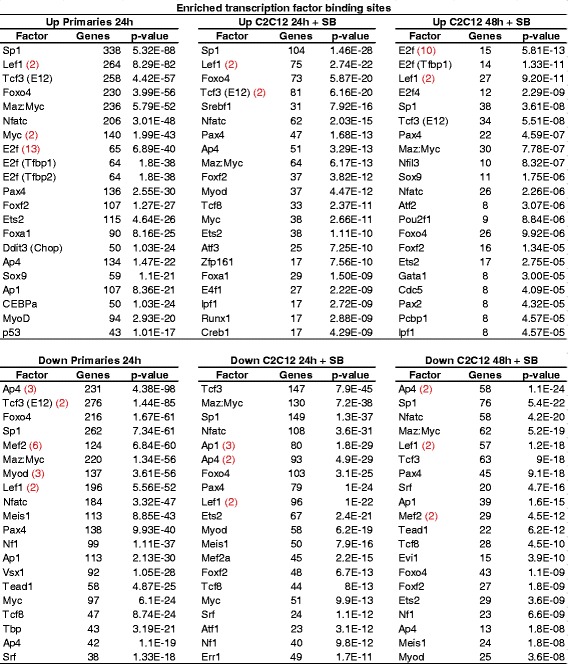
Table showing transcription factor binding sites of p38α-regulated genes. Analyses of DNA-binding elements present in promoters were performed using GSEA. *Red numbers* indicate number of times that the factor appeared in the analysis with different DNA-binding elements, the highest *P* value element is shown. Unknown binding elements have been omitted

In order to identify transcription factor binding sites of genes regulated by p38α, we performed a gene set enrichment analysis (GSEA) in both types of cells and p38α-inhibitory conditions. The analysis showed that genes regulated by p38α have enriched binding sites for different transcription factors, such as Sp1, Lef1, Tcf3/E47, FoxO4, Pax4, MyoD, or NFATc (Fig. 3), and most of them are common in all conditions analyzed (satellite cells 24 h DM, C2C12 24 h DM, and 48 h DM; up- and downregulated). Interestingly, E2F-binding sites are enriched exclusively in p38α-regulated genes in p38α-deficient satellite cells at 24 h DM, and in C2C12 cells at 48 h (but not at 24 h) DM, consistent with the dysregulation of the cell-cycle/proliferation gene program.

### Identification of p38α-chromatin binding sites at the myogenic proliferation-to-differentiation switch

**Fig. 4:**
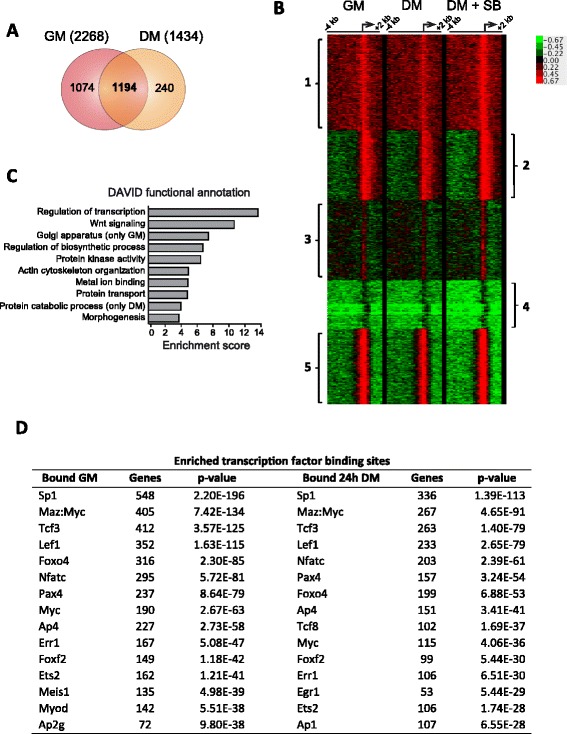
ChIP-on-chip analysis for p38α in myogenic cells. Chromatin immunoprecipitation (ChIP) was performed in C2C12 myoblasts (under different culture conditions) using a p38α-specific antibody. **a** Non-proportional 2-way Venn diagram showing the number of promoters bound by p38α in the two experimental conditions: proliferation (GM) and 24 h of differentiation (24 h DM). **b** Clustering analysis of genes bound by p38α in GM and 24 h DM. Genes were grouped in five clusters according to the pattern of binding along the promoter. Cluster 1 is enriched in transcription factors, developmental regulators, and muscle-related genes. Cluster 2 is enriched in genes involved in bone development, developmental regulators, and transcription factors and cluster 3 includes miscellaneous genes. Cluster 4 is enriched in sensory perception and olfactory receptors whereas cluster 5 is enriched in protein transport, phosphorylation, and cell cycle-related genes. **c** Functional annotation analysis of the Gene ontology (GO) of bound genes in GM and 24 h DM was performed using DAVID. The top annotation clusters are shown according to their enrichment score. Names are based on enriched GO annotations. **d** Table showing transcription factor binding sites of p38α-bound genes. Analyses of DNA-binding elements present in promoters were performed using GSEA. The highest *P* value element is shown. Unknown binding elements have been omitted

To investigate whether p38α regulates global gene transcription at the myoblast proliferation-to-differentiation transition by directly acting at chromatin, we investigated the association of p38α to gene promoters in proliferating (GM) and early differentiating myoblasts (24 h DM) by ChIP-on-chip analysis using a p38α-specific antibody. Following our transcriptome analysis, and to focus on the early events of differentiation and p38α activation, we performed our analysis in C2C12 cells. The obtained ChIP-on-chip data revealed that the binding of p38α is markedly enriched at many gene promoters both in proliferation and early differentiation stages, consistent with its localization in the nuclear compartment in these two conditions (Additional file 3: Figure S2). A stringent analysis unveiled 2268 p38α-bound genes in myoblasts in GM and 1434-bound genes in DM. We detected a high overlap between GM and DM as more than 1000 genes were bound by p38α in both myogenic conditions (Fig. 4a). A general clustering analysis of genes bound by p38α in C2C12 cells identified genes that could be grouped in five clusters according to the pattern of binding along the promoter (Fig. 4b). Interestingly, cluster 1 included many transcription factors, developmental regulators, and muscle-related genes, whereas cluster 5 was enriched in protein transport, phosphorylation, and cell cycle-related genes. GO analysis revealed common p38α-bound genes enriched in positive and negative regulators of transcription, Wnt signaling pathway, actin cytoskeleton organization, and serine/threonine and protein kinase-related genes; genes bound only in proliferative conditions were enriched in Golgi apparatus genes, positive regulators of transcription and further enriched in Wnt signaling molecules. Interestingly, genes bound only in differentiating conditions were enriched in glycoproteins and membrane proteins, and in general, but negative regulators of transcription (positive general transcriptional regulators were not enriched) (Fig. 4c). Moreover, GSEA analysis of transcription factor binding sites in the p38α-bound genes identified enriched binding sites for nearly the same transcription factors found in p38α-regulated genes obtained in the expression microarrays, for example: Sp1, Lef1, Tcf3/E47, FoxO4, Pax4, MyoD, or Nfatc (Fig. 4d). Since some of these transcription factors have been described as phosphorylation substrates of p38 MAPK in different model systems, including Sp1 [32, 33] Tcf3/E47 [12], NFATC4 [34], or E2F4 [35], and the pattern of p38α recruitment to genes is mostly restricted to proximal promoter regions, it is tempting to propose that p38α will likely regulate the myoblast proliferation-to-differentiation transition mainly through these transcription factors.

**Fig. 5:**
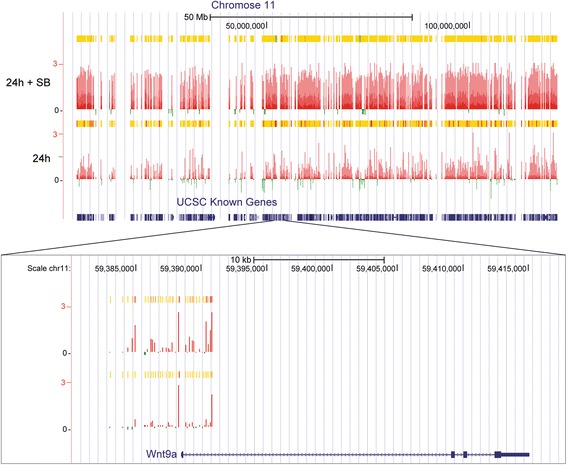
The kinase activity is not required for p38α recruitment to chromatin. p38α-bound promoters in full chromosome 11 and the promoter of Wnt9a, one of the p38α-regulated genes, as an example of the general increased occupancy signal observed on promoters when cells were treated with the SB203580 p38α/β inhibitor. UCSC genome browser with overimposed binding signal is shown

Of note, genome-wide binding analysis of p38α in experimental conditions of myogenic differentiation, where p38α is expressed but its kinase activity is inhibited (C2C12 cells in DM + SB), revealed that the kinase activity was not required for p38α recruitment to chromatin; on the contrary, at the genome level, there was a generally increased occupancy signal on promoters when cells were treated with the p38α/β inhibitor, as exemplified by full chromosome 11 and the promoter of Wnt9a, one of the p38α-regulated genes (Fig. 5). This indicates that recruitment of p38α to certain gene promoters is independent of its kinase activity, although the underlying reason is currently unknown.

### Identification of genes bound and regulated by p38α during early myogenic differentiation

**Fig. 6:**
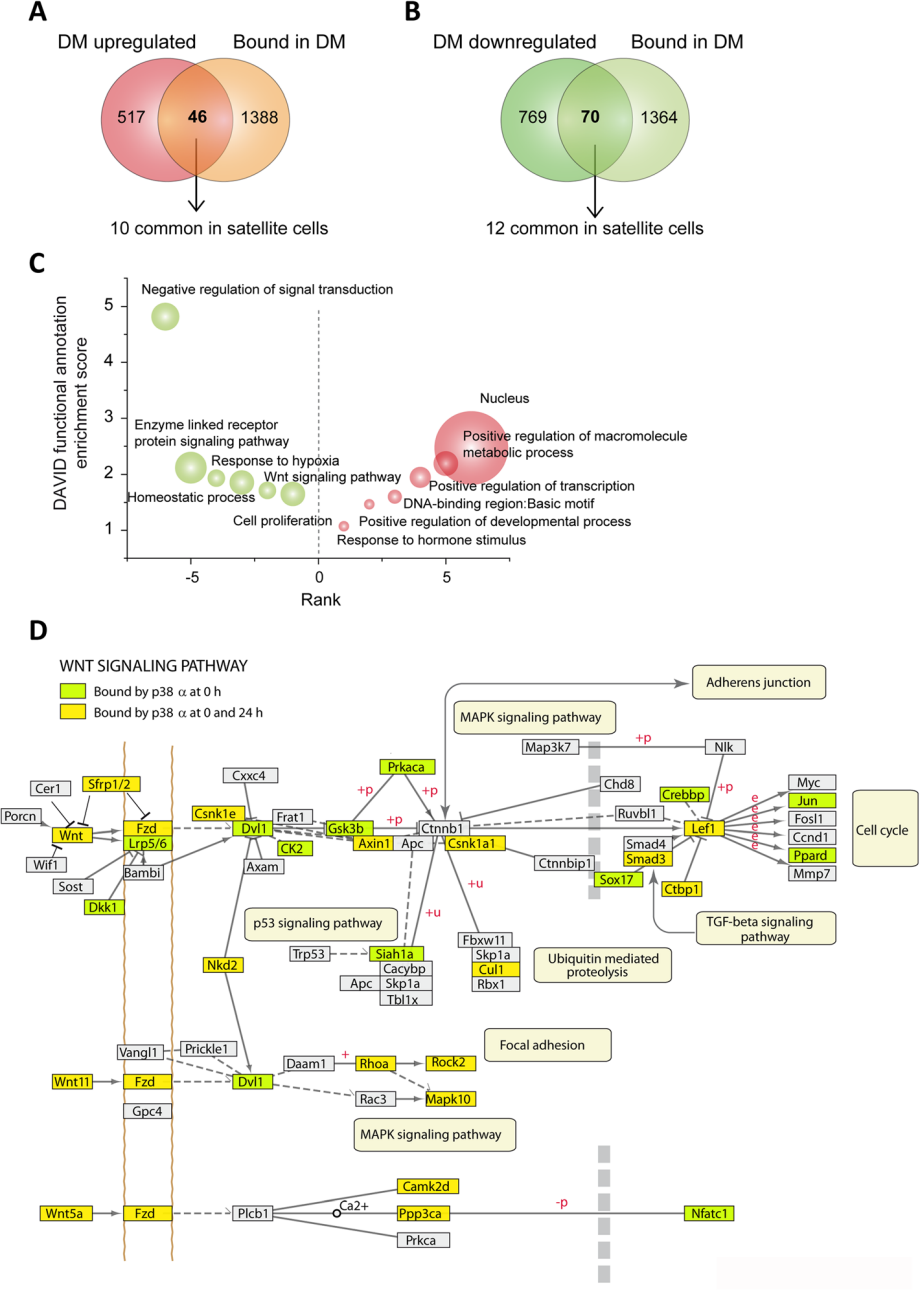
Identification of genes bound and regulated by p38α. **a**, **b** The two data sets (expression array and ChIP-on-chip) were crossed to identify genes bound and regulated by p38α. The non-proportional 2-way Venn diagrams show that, at 24 h of differentiation (DM), p38α binds to the promoter of 46 genes upregulated in SB-treated C2C12 cells (**a**) and to 70 genes downregulated in myoblasts lacking active p38α (**b**). Only 10 and 12 of these bound genes are also upregulated and downregulated, respectively, in p38α-deficient satellite cells. **c** Gene ontology analysis of genes bound and regulated by p38α in C2C12 cells at 24 h in DM. Enriched GO annotations of genes downregulated in SB-treated C2C12 myoblasts are shown in green whereas enriched GO annotations of upregulated genes are shown in red. **d** DAVID analysis of canonical pathways enriched in genes bound by p38α showed that Wnt signaling pathway was one of the main signaling modules regulated by p38α. KEGG pathway is shown with p38α-bound promoters highlighted

To complete the identification of the genes bound and regulated by p38α at the myogenic proliferation-to-differentiation switch, we crossed the two data sets: the expression microarray and the ChIP-on-chip data. We identified 46 genes bound by p38α that were upregulated in SB-treated C2C12 myoblasts, and 70 genes bound and downregulated in similar C2C12 cell culture conditions. Of these, only 10 and 12 genes were also up- and downregulated, respectively, in p38α-deficient satellite cells (Fig. 6a, b). Furthermore, GO analysis of the genes bound and regulated by p38α in differentiating C2C12 cells showed that they are mainly involved in signal transduction pathways, response to hypoxia, homeostatic processes, and cell proliferation. On the other hand, GO analysis of genes bound by p38α and upregulated when p38α activity was chemically inhibited showed enrichment for transcription factors and nuclear receptor coactivators (Fig. 6c). Interestingly, DAVID analysis of canonical pathways enriched in genes bound by p38α highlighted the Wnt signaling pathway as the main signaling module regulated by p38α (Fig. 6d).

**Fig. 7:**
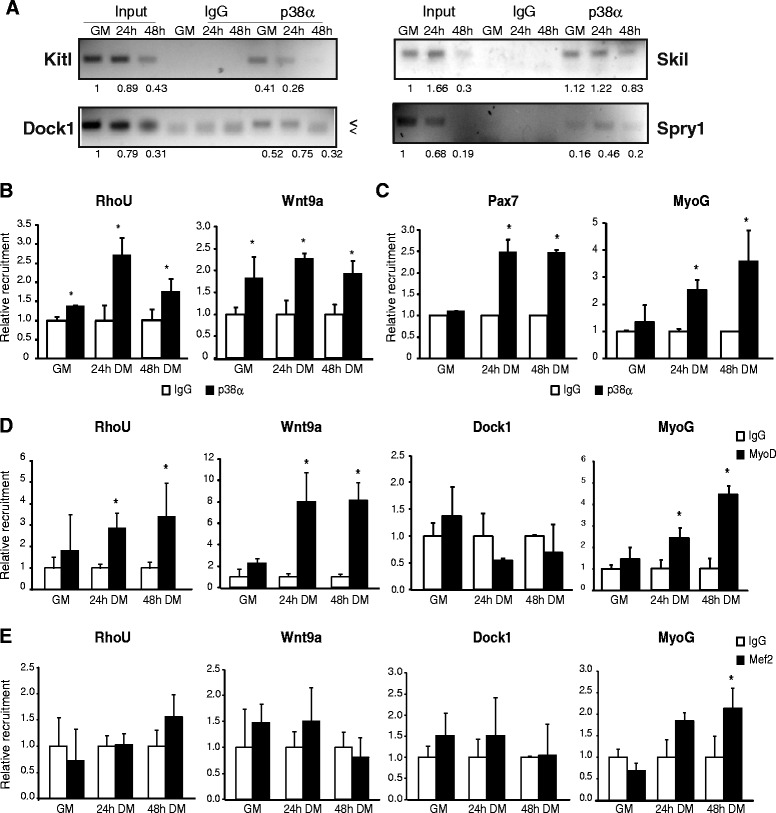
Validation of p38α binding at several identified targets. **a** Proliferating (GM) or differentiating (24 and 48 h DM) C2C12 cells were subjected to ChIP analysis with antibodies to p38α or control rabbit IgG. Immunopurified DNA was subjected to semiquantitative PCR with indicated primers, and the PCR products were then run on an agarose gel. Representative images are shown. Densitometry of the bands was measured using ImageJ and normalized to input. **b**, **c** DNA from C2C12 cells was immunopurified as in (**a**) and then subjected to qPCR analysis with indicated primers. Data show the relative recruitment of p38α at these promoters and represent the mean of three independent experiments ± SEM. Non-parametric Mann–Whitney *U* test was used to assess statistical significance (*P* value <0.05). **d**, **e** Proliferating (GM) or differentiating (24 and 48 h DM) C2C12 cells were subjected to ChIP analysis with antibodies to MyoD, Mef2 or control rabbit IgG and immunopurified DNA was subjected to qPCR analysis with indicated primers

We validated p38α binding to the promoters of genes up- or downregulated after p38 inactivation in myogenic cells belonging to the different GO categories and pathways described above: Wnt signaling pathway (Wnt9a), actin cytoskeleton organization (Dock1), cellular signaling (RhoU, Kitl and Skil), and regulation of transcription (Spry1). This validation was performed through ChIP of cells cultured in three different conditions (GM, 24, and 48 h DM—this latter time-point was included to extend the kinetics of p38α binding), and subsequent semiquantitative or qPCR analysis of the immunopurified DNA (Fig. 7a, b). Moreover, and consistent with previously published results [15, 21], we found that p38α recruitment to Myog and Pax7 promoters is increased upon differentiation (Fig. 7c). Of note, actual recruitment of the muscle regulatory factor MyoD (but not Mef2) to the newly identified p38α targets Wnt9a and RhoU in differentiating C2C12 myoblasts was confirmed by ChIP-qPCR (Fig. 7d, e).

## Discussion

During myogenesis, satellite cells need to cease proliferation and initiate differentiation as a key step to fuse and form multinucleated myotubes. In vivo, this is the central step for the success of the tissue regeneration process in response to injury (i.e., forming new myofibers to repair the damaged muscle tissue). Previous studies had shown that the proliferation-to-differentiation transition of myoblasts (derived from satellite cells or from immortalized myogenic cell lines) requires timely activation of p38α MAPK (reviewed in [3, 4, 7, 9]), being this isoform also needed for proper muscle regeneration in mice [25]. Interestingly, this isoform was also shown to play an important role in muscular dystrophy progression in mice [36]. Here, we show that p38α exerts this myogenic function at least in part via binding and acting at chromatin. By genome-wide localization analysis coupled to gene expression profiling, we found this p38 isoform associated with mammalian loci more frequently than in any study previously published. In particular, p38α associated with chromatin during the myoblast proliferation-to-differentiation transition, and this correlated with the high levels of transcription, supporting the relevance of kinase signaling pathways in directly regulating transcription, in agreement with previous reports [37–40]. At variance with the classical view of p38α as a positive regulator of transcription [12, 41], we also found that the p38α-chromatin binding was not always associated with active transcription during this myogenic transition, since many p38α-bound promoters were transcriptionally inactive or repressed. Thus, p38α is recruited to a large set of myogenic gene promoters to facilitate their activation or repression, hence pointing to more complex regulatory mechanisms than previously anticipated.

How is p38α recruited to muscle loci is not known, but it likely involves interaction with chromatin-regulatory and/or transcription factors, as demonstrated for several stress-induced genes [42]. Of interest, p38α-bound promoters are enriched with binding motifs for several transcription factors, principally Sp1, Tcf3/E47, Lef1, FoxO4, MyoD, and NFATc, which are known to be phosphorylation substrates of p38 MAPK. In skeletal myoblasts, in particular, p38α associates with MyoD and E47 [12, 15] transcription factors, and specific phosphorylation of E47 promotes E47/MyoD dimerization, binding to muscle-specific loci and initiation of gene expression [12]. Likewise, phosphorylation of MEF2 by p38 facilitates both recruitment of MyoD and expression of late myogenic genes, in a feed-forward mechanism [11], and of ASH2L-MLL methyltransferase complex [18, 19]. p38α also phosphorylates Baf60c allowing SWI/SNF-mediated chromatin remodeling at muscle-specific loci [15–17]. Interestingly, p38α was also shown to represses Pax7 gene expression via direct Ezh2 phosphorylation, as an indispensable event for the transition of proliferating myoblasts into their differentiating state [21]. Thus, p38α may help both silence proliferation-associated genes and activate differentiation-promoting genes at this transition, by directly targeting negative and positive epigenetic and/or transcriptional regulators. Because the p38 MAPK homolog Hog1 targets the RNA Pol II machinery and induces chromatin remodeling at stress-responsive loci in yeast [43], it is possible that p38α regulates Pol II-dependent gene transcription in mammalian myogenesis in a similar way. It is worth noting that p38 was also found on a subset of genes independently of its kinase activity. Our work adds to several recent studies of protein kinases that bind and act on chromatin: extracellular signal-regulated kinase (ERK), cJun N-terminal kinase (JNK), the MSK, AMPK, and Dyrk1A [37, 38, 44–46]. This study also complements the previously described association of p38/Hog1 to osmostress genes to mediate adaptation to extracellular changes in mammals and yeast [26, 27, 42].

Of interest, we observed slower muscle differentiation kinetics in C2C12 myoblasts compared to satellite cells in culture; although the underlying causes for this difference are not clear, they may relate to the distinct nature of both cell types (freshly isolated cells versus immortalized cells) and the different velocity of p38α activation in both cell types (see Fig. 1a). Whether the expression of the identified p38α-regulated late genes depends on the early ones, according to the proposed temporal regulation of muscle gene expression through a MyoD-mediated feed-forward circuit involving p38 MAPK [11], is not yet known.

Finally, although the nature of p38α-regulated genes encompasses several categories, DAVID analysis of canonical pathways enriched in genes bound by p38α showed the Wnt pathway as the main signaling module regulated by p38α. Consistent with this, MyoD was found to be recruited to the newly identified p38α target Wnt9a, in addition to muscle-specific gene promoters, as expected. This is worth highlighting based on the reported relevant role of canonical and non-canonical Wnt signaling in distinct satellite cell functions in vivo (expansion, switching from proliferation to differentiation or cell motility) [47–49]. Taken together, this study increases our understanding on how p38 MAPK regulates gene expression during myogenesis, and greatly enlarges the number and nature of target genes downstream of p38α activation. Notably, through this analysis, we have become aware of new categories of genes whose repression depends on p38α both in proliferating and differentiating conditions, expanding, yet complicating, the function of p38α as a negative regulator of muscle gene transcription. It is possible that comparable principles of chromatin binding and activity might be relevant for other kinases.

## Conclusions

Collectively, through this study, we demonstrate association and action of p38α MAPK throughout chromatin uncovering new classes of target genes during the transition of myoblasts from proliferation to differentiation. The recruitment of p38α to large sets of gene promoters appears to facilitate their activation or repression during this process. This is consistent with this MAPK isoform being a transcriptional regulator.

## Additional files

Additional file 1: Table S2. List of primers used in the study. (XLSX 12 kb) Additional file 2: Table S1. List of genes regulated and bound by p38α. (XLSX 686 kb) Additional file 3:
**Supplementary Figures 1 and 2 and legends. Figure S1.** Relative expression of p38α and p38β in C2C12 cells. The expression of p38α and p38β was measured by qPCR in differentiating C2C12 cells. Values are mean ± SEM of three independent set of samples. **Figure S2.** Nuclear p38α remains constant in proliferating and differentiating myoblasts. Expression of p38α in C2C12 cells in GM and 24 h in DM: p38α (green) and DAPI (blue). Nuclear to cytoplasm fluorescence ratio was calculated using integrated density values in ImageJ software with DAPI as nuclear boundary. Scale bar = 25 μm. (PDF 7595 kb)

## Abbreviations

BAF60c: BRG1/BRM-associated factor 60 variant c
ChIP: chromatin immunoprecipitation
DAPI: 4′,6-diamidino-2-phenylindole
DM: differentiation medium
Dock1: dedicator of cytokinesis
FoxO4: forkhead box protein O4
GM: growth medium
GO: gene ontology
GSEA: gene set enrichment analysis
Lef1: lymphoid enhancer-binding factor 1
MAPK: mitogen-activated protein kinase
MEF2: myocyte enhancer factor 2
MRFs: myogenic regulatory factors
NFATc: nuclear factor of activated T cells
Pax4: paired box gene 4
RT-qPCR: reverse transcriptase quantitative polymerase chain reaction
Spry1: protein sprouty homolog 1
Tcf3: transcription factor 3 (E2A immunoglobulin enhancer-binding factors E12/E47)
Wnt9a: wingless-type MMTV integration site family, member 9A
WT: wild type
