# Supplementary material for: Chromatin-wide and transcriptome profiling integration uncovers p38α MAPK as a global regulator of skeletal muscle differentiation
Source: Skelet Muscle. 2016 Mar 15;6:9. doi: 10.1186/s13395-016-0074-x (PMC4791895; doi:10.1186/s13395-016-0074-x)
Supplement: Additional file 3: — Supplementary Figures 1 and 2 and legends. Figure S1. Relative expression of p38α and p38β in C2C12 cells. The expression of p38α and p38β was measured by qPCR in differentiating C2C12 cells. Values are mean ± SEM of three independent set of samples. Figure S2. Nuclear p38α remains constant in proliferating and differentiating myoblasts. Expression of p38α in C2C12 cells in GM and 24 h in DM: p38α (green) and DAPI (blue). Nuclear to cytoplasm fluorescence ratio was calculated using integrated density values in ImageJ software with DAPI as nuclear boundary. Scale bar = 25 μm. (PDF 7595 kb) [file 13395_2016_74_MOESM3_ESM.pdf]

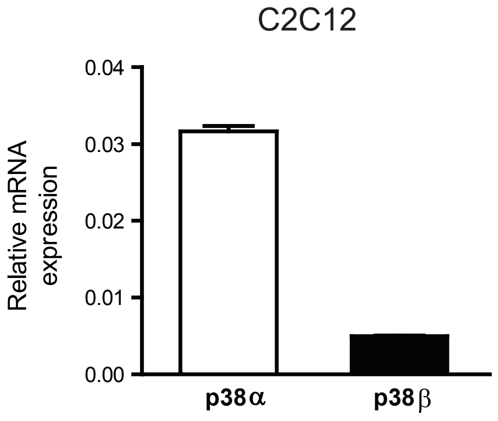
C2C12
Relative mRNA expression
0.04
0.03
0.02
0.01
0.00
p38α
p38β

Figure S1

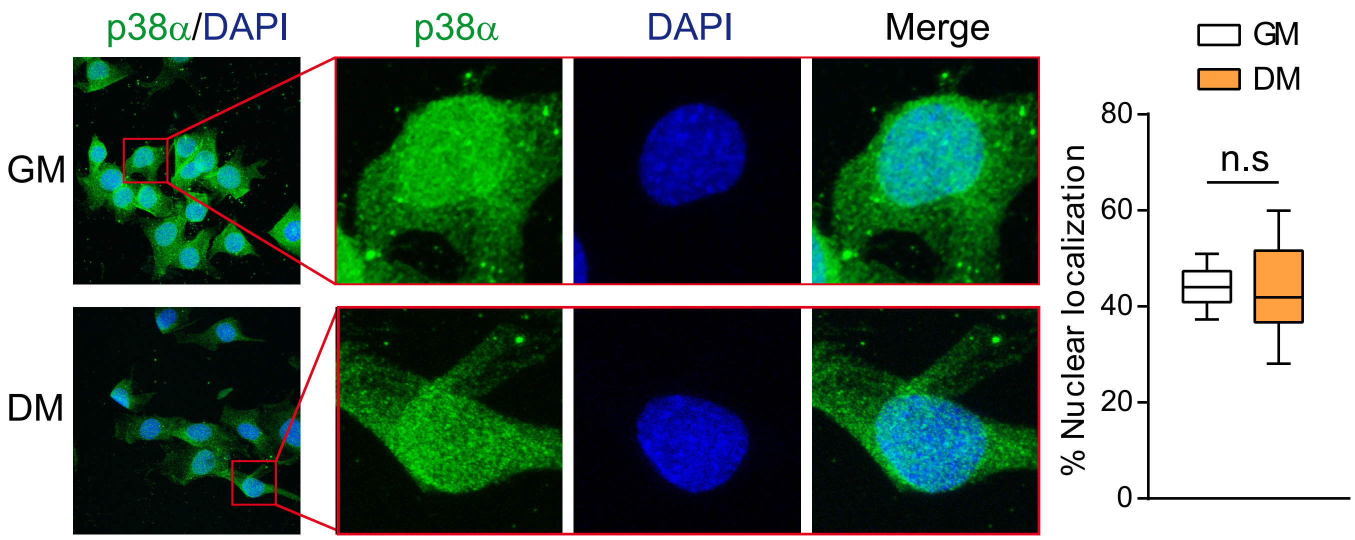
p38α/DAPI
p38α
DAPI
Merge
GM
DM
GM
DM
80
60
40
20
0
% Nuclear localization
n.s

Figure S2

Supplementary Figure legends

Fig S1. Relative expression of p38α and p38β in C2C12 cells. The expression of p38α and p38β was measured by qPCR in differentiating C2C12 cells. Values are mean ± SEM of 3 independent set of samples.

Fig S2. Nuclear p38α remains constant in proliferating and differentiating myoblasts. Expression of p38α in C2C12 cells in GM and 24h in DM: p38α (green) and DAPI (blue). Nuclear to cytoplasm fluorescence ratio was calculated using Integrated Density values in Image J software with DAPI as nuclear boundary. Scale Bar = 25 µm.
